# Methane storage in nanoporous material at supercritical temperature over a wide range of pressures

**DOI:** 10.1038/srep33461

**Published:** 2016-09-15

**Authors:** Keliu Wu, Zhangxin Chen, Xiangfang Li, Xiaohu Dong

**Affiliations:** 1 The Department of Chemical and Petroleum Engineering, University of Calgary, Alberta T2N1N4, Canada; 2 Key Laboratory for Petroleum Engineering of the Ministry of Education, China University of Petroleum, Beijing 102249, China

## Abstract

The methane storage behavior in nanoporous material is significantly different from that of a bulk phase, and has a fundamental role in methane extraction from shale and its storage for vehicular applications. Here we show that the behavior and mechanisms of the methane storage are mainly dominated by the ratio of the interaction between methane molecules and nanopores walls to the methane intermolecular interaction, and a geometric constraint. By linking the macroscopic properties of the methane storage to the microscopic properties of a system of methane molecules-nanopores walls, we develop an equation of state for methane at supercritical temperature over a wide range of pressures. Molecular dynamic simulation data demonstrates that this equation is able to relate very well the methane storage behavior with each of the key physical parameters, including a pore size and shape and wall chemistry and roughness. Moreover, this equation only requires one fitted parameter, and is simple, reliable and powerful in application.

Rapidly rising population from today’s 7 billion to estimated 9 billion by 2050^1^ will place tremendous energy demand around the world^2^, increasing from about 12 billion tonne oil equivalents (t.o.e.) in 2009 to 18 billion t.o.e. by 2035 predicated by the International Energy Agency based in Paris^3^. With the unavoidable depletion of conventional petroleum-based fuels and their serious greenhouse effect^4^,^5^, natural gas, consisting mainly of methane, is an attractive and great potential “bridge fuel” during transition to carbon-free fuels^6^ thanks to its abundant reserves^7^,^8^,^9^,^10^, wide distribution^11^, low CO_2_ emission^12^ and economic efficiency^4^.

During the past decade, horizontal drilling and hydraulic fracturing make successful extraction of natural gas from shale economically feasible^13^. Shale, characterized by abundant nanopores, including organic kerogen pores and inorganic pores, hosts free gas and adsorbed gas because of large internal surface areas^14^. In addition, these nanopores are irregular in their cross sections, including bubble-like, elliptical and faveolated shapes^15^. Therefore, they lead to significant challenges in shale gas reserve estimation, extraction and production predication. On the other hand, increased improvements in shale gas extraction have also driven renewed interest in natural gas application in transportation^16^. However, a main technological barrier for the widespread use of natural gas as an alternative vehicular fuel is its relatively low volumetric energy density compared with gasoline^17^,^18^. Adsorbed natural gas may more potentially overcome this barrier compared with conventional storage technologies, including compressed natural gas and liquefied natural gas^4^,^5^,^7^,^16^,^17^. The adsorbed natural gas technology may store more natural gas at much lower pressure with lower costs and better safety^18^,^19^. To accelerate the vehicular application of nature gas, the US Department of Energy has set an ambitious target for a volumetric storage capacity of 350 cm^3^ (STP) cm^−3^ (adsorbent) and a gravimetric storage capacity of 0.5 g (CH_4_) g^−1^ (adsorbent) at operational conditions^20^,^21^. It is a formidable challenge for chemists and material scientists to develop a novel technology to achieve this target^17^,^18^.

Important research has been conducted about adsorbed natural gas in nanoporous materials: zeolites^22^,^23^,^24^, zeolitic imidazolate frameworks^25^,^26^,^27^,^28^,^29^, nanoporous carbons^30^,^31^, porous organic polymer networks^32^, covalent organic frameworks^33^, and metal-organic frameworks (MOFs)^34^,^35^,^36^,^37^. MOFs, for example, a relatively new family of nanoporous materials, have become very important in gas storage application because they can be designed at the atomic scale and tailored systematically in their chemical composition, functionality, and pore size to maximize natural gas storage capacity^10^,^38^. Even with all the research, the storage of methane molecules in nanoporous materials is still a fundamental and long-standing challenging issue for its applications in both transportation and the above mentioned shale gas reserve estimation, extraction, and prediction.

To better understand and manipulate the methane storage in nanoporous materials, it is key to shed light on its underlying mechanisms. The methane storage behavior in nanoporous materials arises primarily from the interaction between methane molecules and nanopores walls^30^, and is also influenced by the intermolecular interaction of methane, especially in the case of high pressure^39^. A nanopore size^5^,^40^ and shape^12^,^38^, wall chemistry^5^,^7^,^18^ and roughness^41^, and operational conditions including pressure^16^ and temperature^42^ induce a varying ratio of the two interaction strengths mentioned and influence the methane storage behavior in nanopores. Similarly, the methane storage behavior of nanoporous materials is also controlled by porosity^43^,^44^, nanopore size distribution^45^, nanopore connectivity^24^, and specific surface areas^31^,^44^,^46^. Methane is mainly stored in micropores (<2 nm) or mesopores (2–50 nm) at supercritical temperature (above 273.15 K) over a wide range of pressures (up to 50 MPa) in shale gas reservoirs and vehicular applications. Under such extreme surrounding conditions, the methane storage behavior is significantly complex, and has not been studied to our knowledge. Moreover, understanding and manipulating the methane storage behavior is crucial to extract successfully methane from shale formation^11^ and move it to the market in vehicular applications^5^,^43^,^47^.

The gas storage behavior in nanoporous materials can be investigated through experiments^5^,^14^,^48^,^49^,^50^, molecular dynamics (MD) simulations^8^,^11^,^46^,^51^, mathematical models^47^,^52^,^53^, and combining these methods^54^,^55^. Experiments are the closest to reality; furthermore, with rapid advancement in experimental equipment and technologies today, it is possible by direct observation to reveal some undiscovered and underlying mechanisms, such as adsorption sites determination^49^ and adsorbed gas molecules structures and ordering^50^. However, experiments are expensive, time-consuming^47^, and hard to identify an effect of a single key parameter^30^. MD simulations and mathematical models, inevitable complements to experiments, can predict and validate experimental results, and even reveal undiscovered experimental phenomena^56^,^57^. Although recent advances have greatly enhanced computational performance, due to a large number of atoms and force field calculations involved in modeling the gas storage behavior, MD simulations are still computationally expensive and time-consuming, with each study case requiring a separate modeling, compared with mathematical models^30^. In contrast, a practical mathematical model, based on some assumptions and approximations, not only provides instantaneous calculation results and identifies the effect of each key physical parameter but also yields general predictions and observations^47^,^52^,^53^. These advantages of mathematical models are especially remarkable in modeling the gas storage behavior in nanoporous materials. For example, the methane storage behavior in complex nanoporous shale varies during a depressurized development process of shale gas reservoirs; when conducting a numerical simulation for production prediction, millions of computational grid blocks need to participate in the simulation and can be very expensive.

In this work, we have developed a computationally efficient equation of state (EOS) for methane in nanoporous materials at supercritical temperature over a wide range of pressures. This equation takes into account the interaction between methane molecules and nanopore walls, methane intermolecular interaction, and the varying ratio of both interactions with pressure. In particular, the equation is able to describe the methane storage in nanoporous shale at pressure up to 50 MPa where its storage behavior is significantly different from its counterpart at low pressure, such as the methane storage in vehicular application. Furthermore, an application of this equation requires only one parameter that can be determined by fitting experimental data or MD simulation results. Due to its simple and robust nature, it is readily extended to other gases. We use this equation to investigate the methane storage behavior in nanoporous materials at supercritical temperature over a wide range of pressures, elucidate the effect of each nanoporous material property (e.g., a nanopore size and shape, and wall chemistry and roughness), and improve an understanding of the correlation between the gas storage behavior and these nanoporous material properties. The work provides an effective, reliable and powerful tool for modeling the shale gas storage behavior, and screening and designing nanoporous materials for methane storage in vehicular applications.

## Theoretical background

**Figure 1:**
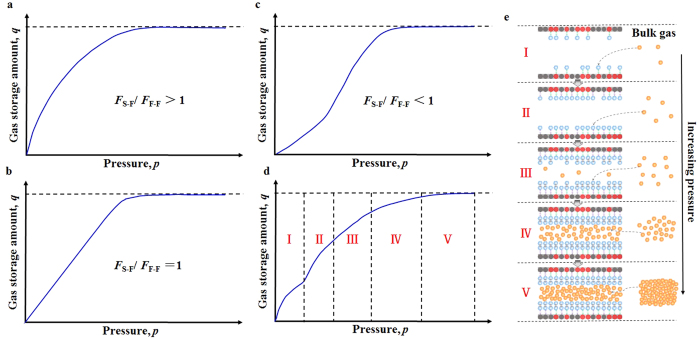
Schematic representation of gas storage at supercritical temperature in nanopores with different interactions.

Gas storage mechanisms determine the characteristics of gas storage behavior in nanoporous materials. Some of the gas storage mechanisms can be revealed by advanced experimental technologies today; for example, the gas storage properties (adsorbed gas density and the number of layers) can be characterized by the Nuclear Magnetic Resonance (NMR)^14^, and even the adsorption sites and the ordering of adsorbed gas molecules can be detected by *in situ* small-angle X-ray scattering^49^,^50^. The gas storage mechanisms are mainly dominated by the ratio of the interaction between gas molecules and nanopores walls to the gas intermolecular interaction^8^,^39^,^58^,^59^. For gas storage in nanopores with walls having homogeneous chemical and physical properties, while the interaction between gas molecules and nanopores walls dominates (*F*_S-F_/*F*_F-F_ > 1, where *F*_S-F_ is the interaction force between gas molecules and nanopores walls and *F*_F-F_ is the gas intermolecular interaction force.), gas storage amount first increases sharply with pressure in the low pressure region, then increases slowly in the relatively high pressure region^11^,^60^, and finally becomes constant due to the storage saturation limited by the space available for gas molecules^61^ (see Fig. 1a). Similar but different in the low pressure region^5^, the gas storage behavior for *F*_S-F_/*F*_F-F_ = 1 and *F*_S-F_/*F*_F-F_ < 1 is shown in Fig. 1b,c, respectively. Both interactions arise from the van der Waal forces, including a Keesom force (two permanent dipoles), a Debye force (a permanent dipole and a corresponding induced dipole), and a London dispersion force (two instantaneously induced dipoles)^62^. For gas storage in nanopores with walls having heterogeneous chemical and physical properties, in the low pressure region, gas molecules first prefer to adsorb at strong adsorption sites induced by the Keesom force (see the I region in Fig. 1d,e) or at small groves due to the roughness of a wall (not shown in Fig. 1d,e), and then adsorb at the relatively weak adsorption sites induced by the Debye force (see the II region in Fig. 1d,e). When pressure increases to the middle pressure region, the weak London dispersion force exerted by the adsorbed gas molecules dominates the gas storage behavior^49^,^51^ because the strong and relatively weak adsorption sites have been largely occupied by the adsorbed gas molecules (see the III region in Fig. 1d,e). When the pressure continues to increase to the relatively high pressure region, the gas storage behavior in nanopores is somewhat similar to that in the bulk gas phase due to the dominance of free gas intermolecular interactions (see the IV region in Fig. 1d,e). Finally, the gas storage amount becomes almost unvarying in the high pressure region due to the storage saturation, and is lower than that of the bulk gas phase (see the V region in Fig. 1d,e).

For nanoporous materials for methane storage in vehicular application, their working capacity (also called usable capacity or deliverable capacity), generally defined as the difference in the methane storage amount between 0.5 MPa and 6.5 MPa, is more key than the total storage capacity^5^. To maximize the working capacity, we optimize the number and distribution of the strong and weak adsorption sites on walls to achieve a relatively low methane storage amount in the low pressure region (< 0.5 MPa) and a very high methane storage amount in the relatively high pressure region (up to 6.5 MPa)^7^,^18^,^63^. This is because with increasing pressure, the attractive interaction between gas molecules and the weak adsorption sites gradually becomes to play a more important role (see the II region in Fig. 1d,e). For the methane storage at extremely high pressure (up to 50 MPa) in nanopores of shale gas reservoirs, compared with the bulk gas phase, its storage amount is less (see the V region in Fig. 1d,e) due to the limited available space in nanopores; furthermore, the interval between the first adsorption layer and a wall, about 3.575 Å consistent with the Lennard-Jones (LJ) parameters^11^ (see Fig. 1e), also reduces the available space. In addition, the interaction between methane molecules and the walls also decreases a methane storage amount due to the dominance of the methane intermolecular interaction at high pressure. However, it can enhance the methane storage amount due to its dominance at relatively low pressure; more explanation is given below. Thus, a powerful EOS for methane must capture these unique phenomena in nanopores.

## Results

### Capturing confinement effects

**Figure 2:**
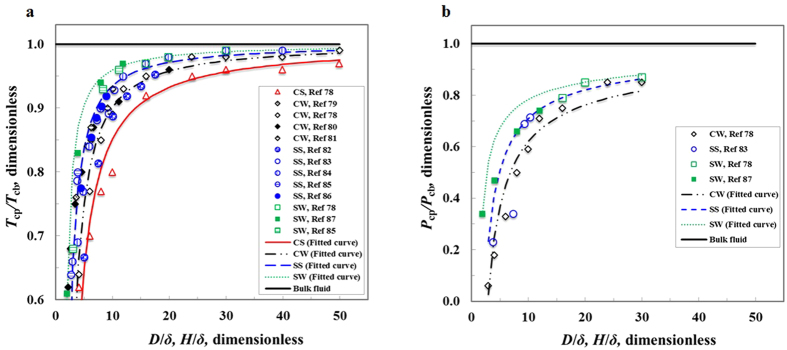
Dependence of critical properties (all reduced by the corresponding bulk values) on nanopore size.

Gas thermodynamic properties in nanopores are significantly different from the corresponding bulk gas properties^64^,^65^,^66^,^67^,^68^,^69^,^70^,^71^,^72^,^73^,^74^. These unique and interesting phenomena are induced, on one hand, by a geometric constraint limiting the gas molecules number^52^, and, on the other hand, by the non-negligible van der Waals forces arising from the interaction between gas molecules and walls in nanoporous materials^75^. Bulk gas molecules move randomly without specific orientation and direction, while the gas molecules confined in nanopores have a relatively well-ordered and layered structure in the axial direction (see Fig. 1e) induced by the geometric constraint and the van der Waals forces exerted by nanopores walls. These structural differences cause changes in gas thermodynamic properties, especially in the critical properties including the critical temperature and critical pressure. The critical temperature and critical pressure both decrease as the nanopores size decreases, even with absence of the van der Waals forces^52^. In addition, the van der Waals forces influence and complicate the varying behavior of the critical properties. The critical properties are dependent on several parameters, such as nanopores sizes and geometry, wall physical and chemical properties, and gas properties^76^ because these factors influence the geometric constraint or the van der Waals forces mentioned above. The varying extents of critical properties, as the key parameters in modeling gas storage in nanoporous materials, can be determined by MD simulations and fitted by data sets (see Fig. 2), and the critical properties of the confined gas calculated by the fitting equations (Equations S1–S7 in Supplementary Methods) can be applied in calculations of the EOS developed below. Generally, for gas confined in nanopores with the same nanopores geometry, the changes of the critical properties are more noticeable for the stronger van der Waals forces and the smaller pores size; for gas confined in nanopores with the same nanopores size and van der Waals forces, the changes are more noticeable in cylindrical nanopores compared with slit nanopores (see Fig. 2). More details about confinement effects elucidated can be found in Supplementary Discussions.

### Characterizing microscopic descriptors of different interactions

To depict exactly the gas storage behavior in nanoporous materials, the macroscopic properties of gas storage must be linked to the microscopic properties of a gas molecules-wall system^61^. The key descriptors of gas storage in nanoporous materials include the strength of the gas intermolecular interaction and the strength of the gas molecule-wall interaction.

In the low pressure regions (the corresponding I and II regions in Fig. 1d,e), the gas storage behavior is mainly controlled by the strength of the gas molecule-wall interaction, which can be characterized by the Henry law equilibrium constant^88^:


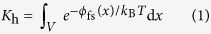


where *K*_h_ is the Henry law equilibrium constant; *V* is the nanopore volume; *ϕ*_fs_(*x*) is the potential of the gas molecule-wall system; *x* is a coordinate; *k*_B_ is the Boltzmann constant; *T* is temperature.

For cylindrical pores, the well-known hypergeometric potential of a gas molecule-wall system is^89^


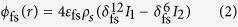


where *ε*_fs_ and *δ*_fs_ are the LJ gas molecule-wall well depth and collision diameter, respectively; *ρ*_s_ is the wall atomic density; *r* is the radial coordinate. *I*_1_ and *I*_2_ are both functions of a nanopore diameter and more details about their calculations can be found in Supplementary Methods.

For slit pores, the potential of a gas molecules-wall system following the Steele potential is^90^,^91^


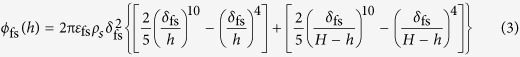


where *h* is the distance away from a wall.

As shown in Equations (2) and (3), a gas molecule interacts with the nearest atom of a wall, and the interaction strength is characterized by *ε*_fs_. In addition, a gas molecule also interacts with all atoms of a wall within a certain cutoff distance, and these multiple interactions are characterized by the surface atomic density *ρ*_s_. In the case of wall roughness, the interaction is further augmented and characterized by an increasing *ε*_fs_ in our work (see Supplementary Tables S1–3). Thus, the gas storage behavior in the low pressure region mainly depends on three physical parameters: gas molecule-wall well depth, wall atomic density, and wall roughness. It is noted that Equations (2) and (3) are invalid in modeling interactions between methane molecules and walls of MOFs, porous organic polymer networks and covalent organic frameworks, while they are able to successfully capture the interactions between methane molecules and walls of activated carbon pores, silica pores and nanoporous shale.

When pressure increases (above the middle pressure region corresponding to the III and IV regions in Fig. 1d, e), the gas intermolecular interaction gradually becomes strong, non-negligible in contribution of the gas storage behavior, and is determined by the intrinsic microscopic properties of gas molecules, such as their shape, polarizability, and permanent electric moments^61^. The interaction strength can be characterized by the equilibrium constant for gas:


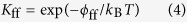


where *ϕ*_ff_ is the potential of a gas molecule-molecule system and expressed as^61^


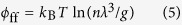


where *n* is the methane density, *λ* is the de Broglie thermal wavelength of methane, and *g* is the spin degeneracy of methane. More details about their calculation results can be found in Fig. S1a.

### Developing EOS for methane in nanoporous materials

A practical, reliable and powerful EOS for methane in nanoporous materials will not only reduce the number of fitted parameters but also capture the key methane storage mechanisms in different pressure regions. In the present work, we develop an EOS with only one fitted parameter, and this EOS links the methane storage behavior and the microscopic properties including the varying critical properties, the strength of the gas intermolecular interaction, and the strength of the gas molecule-wall interaction as elucidated above.

In the relatively high pressure region (the IV region in Fig. 1d,e), the gas intermolecular interaction dominates the gas storage behavior, and this process can be quantitatively depicted by an appropriate EOS^61^. For methane at supercritical temperature, the Redlich-Kwong (RK) EOS^92^ is chosen as the basic equation for developing our EOS, due to its excellent prediction for light hydrocarbons in a supercritical region^93^:


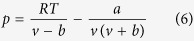


where *p* is the pressure; *R* is the universal gas constant; *v* is the gas molar volume; *a *= 0.42748*R*^2^*T*_c_^2^*T*_r_^−0.5^/*p*_c_ is an attraction parameter; *b *= 0.08664 *RT*_c_/*p*_c_ is a repulsion parameter; *p*_c_ is the critical pressure; *T*_c_ is the critical temperature; *T*_r_ is the reduced temperature.

In the relatively low pressure regions (the I, II and III regions in Fig. 1d,e), the interaction between gas molecules and walls is non-negligible, and dominates the gas storage behavior in the I and II regions. This interaction depends on the nanopore size and shape, and wall chemistry, atomic density and roughness. The gas storage behavior is mainly controlled by the ratio of the interaction between gas molecules and walls to the gas intermolecular interaction, which can be characterized by using the microscopic descriptors of both interactions derived above (Equations (1) and (4)):


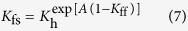


where *A* is a positive fitted parameter for a certain gas-nanoporous material system and is obtained by fitting the experimental or MD data.

As shown in Equation (7), the ratio *K*_fs_ is a function of pressure and temperature. At a certain temperature, when pressure increases to the very high region, *K*_fs_ gradually becomes small and finally approaches 1, indicating that the contribution of the interaction between gas molecules and walls gradually becomes less and finally negligible, while the contribution of the gas intermolecular interaction gradually becomes great and finally dominates, which is similar to a bulk gas; in contrast, when pressure decreases to the very low region, *K*_fs_ gradually becomes large and finally approaches *K*_h_, indicating that the contribution of the interaction between gas molecules and walls gradually dominates and can be quantified by the Henry law equilibrium constant at very low pressure (see Supplementary Fig. S1). At a certain pressure, when temperature is below a certain value, gas molecules do not have enough kinetic energy to overcome the strength of the van der Waals forces exerted by walls^88^ so *K*_fs_ is large and the contribution of the interaction between gas molecules and walls dominates; in contrast, when temperature increases above a certain value, the gas molecules have sufficient kinetic energy to escape from the wall attraction field in the vicinity of a wall^88^ so *K*_fs_ is small and the contribution of the interaction between gas molecules and walls gradually becomes less and finally negligible.

In the extremely high pressure region (the region V in Fig. 1d,e), the gas storage is at saturation due to the geometric constraint^61^ and the van der Waals forces arising from the interaction between walls and gas molecules, and can be characterized by the varying critical properties of methane in nanoporous materials as mentioned above.

To cover these different methane storage mechanisms in different pressure regions, the RK EOS needs to be modified as follows:


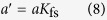



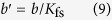


In addition, it is noted that the critical temperature *T*_c_ and critical pressure *p*_c_ for gas confined in nanoporous materials are both functions of a pore size and shape, and wall chemistry, atomic density and roughness as elucidated above, and thus the confinement effects are also considered in this modified equation (our EOS) by substituting the critical temperature *T*_c_ and critical pressure *p*_c_ of the confined gas into the attraction parameter *a* and repulsion parameter *b* in Equations (8) and (9).

Comparing our EOS with the RK EOS, we see that our EOS makes two important improvements. First, the prediction of pseudo-liquid behavior (at supercritical temperature) is more accurate because the repulsion parameter 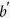 is a molecular volume of the confined gas, which approaches a limiting value at extremely high pressure, and physically represents the repulsive component of pressure at the molecular scale. In order to consider the effects of different interactions and the geometric constraint, 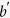 should be described through “*b”* divided by “*K*_fs_”, as shown in Equation (9). Second, the prediction of nonideal behavior is improved because the attraction parameter *a*' accounts for the nonideal behavior of the confined gas storage and physically represents the attractive component of pressure. Similarly, to consider the effects of different interactions and the geometric constraint, it should be expressed through “*a*” multiplied by “*K*_fs_”, as shown in Equation (8). It is noted that our EOS is the same as the RK EOS in application.

**Figure 3:**
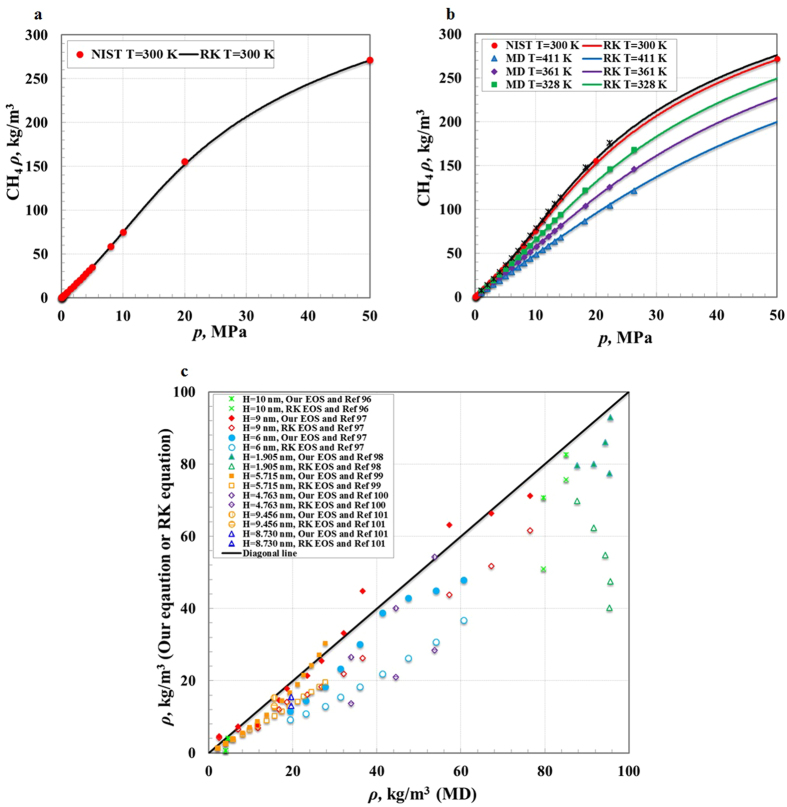
Comparisons of the results calculated by RK EOS and our EOS with data from NIST and MD simulation.

To validate our EOS for methane in nanoporous materials, we utilize the experimental and MD results available in the literature to compare with those calculated by our EOS. First, the chosen basic equation (RK EOS) is reliable in modeling the bulk gas storage at supercritical temperature because of its excellent agreement with data collected by the National Institute of Standards and Technology (NIST)^94^ (see Fig. 3a). Second, MD simulation data is accurate, and can be used to validate our EOS, because the MD simulation data^95^ matches very well with those calculated by the RK EOS (see Fig. 3b). Third, the results obtained by our EOS are consistent with the MD simulation data for gas storage in nanoporous materials^96^ (see Fig. 3c). Despite a variety of nanopore sizes, and wall chemistry, atomic density and roughness, our EOS is validated to be sufficiently accurate in modeling the gas storage behavior in nanoporous materials at supercritical temperature over a wide range of pressures.

Our EOS has the advantages of simplicity and accuracy because there is only one fitted parameter and it captures the key methane storage mechanisms in modeling shale gas storage. In addition, it can quantitatively characterize the relationships between each of property variables and the gas storage behavior in nanoporous materials. Thus, we can use it to screen efficiently the existing nanoporous materials and to sketch an image of the ideal and optimal nanoporous materials for maximizing the gas storage amount.

It is noted that we choose simple pore geometries (cylindrical and slit pores) as nanopore models for deriving the analytical expressions for the varying critical properties and the strength of the gas molecule-wall interaction as elucidated above. In reality, it is impossible to represent real nanoporous materials with perfect cylindrical or slit pores. We have developed a method to represent a real nanoporous material by an assembly of cylindrical and slit pores, guaranteeing that a simple pore model has an equivalent nanopore size possessing the same gas storage behavior as the real nanoporous material. We will show that the simple pore model can be applied to reproduce very well the experimental results of gas storage behavior in a real nanoporous material. We point out that the developed EOS is only valid in modeling the methane storage in activated carbon pores, silica pores and nanoporous shale with the van der Waals interaction. A further study needs to be conducted in extending this EOS to other kinds of porous materials, especially MOFs due to their unique chemical environments.

## Discussions

**Figure 4:**
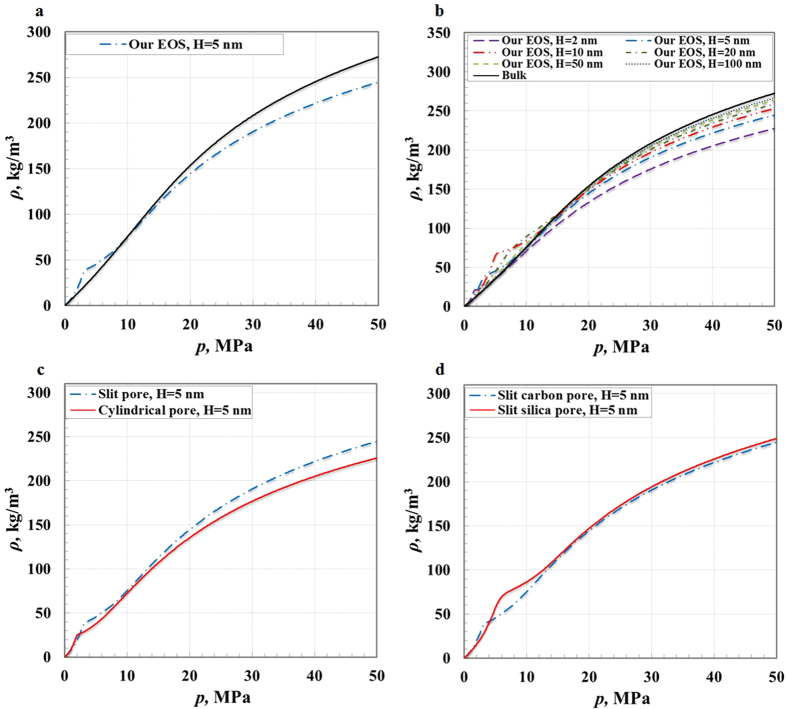
Methane storage behavior in nanopores.

Figure 4 shows that the methane storage amount increases with an increasing pressure in nanopores. However, the methane storage behavior varies in different pressure regions (see Fig. 4a). In the low pressure region, the methane storage amount is larger compared with bulk methane due to the dominance of the interaction between methane molecules and walls that has a positive influence. In the high pressure region, it is smaller compared with bulk methane, because the methane intermolecular interaction dominates and the interaction between methane molecules and walls becomes a negative influence. In the middle pressure regions, it is similar to bulk gas because of the almost equal contributions of both interactions. In addition, the geometric constraint always has a negative influence on the methane storage amount in all pressure regions. As the nanopore size decreases, the interaction between gas molecules and walls gradually becomes strong, and the methane storage capacity increases at low pressure and decreases at high pressure. Moreover, smaller nanopores are of quicker saturation of methane storage due to the overlapping of interactions with opposite walls^11^ (see Fig. 4b). A pore shape plays a crucial role in the gas storage behavior; with the same pore size, compared with slit pores, the gas storage capacity in cylindrical pores is higher at low pressure, and lower above a relatively high pressure, due to the difference of the interactions of methane molecules and walls for both types of pores (see Fig. 4c). In addition, nanopore wall energy sites also control the interaction of methane molecules and walls, and influence the gas storage behavior (see Fig. 4d). Different energy sites on nanopore walls have significant differences in polarizing the methane molecules, and result in differences in the strengths of the interaction between methane molecules and walls. These energy sites arise from many sources, such as functional groups (type, number and position), and wall atomic density and roughness (see Supplementary Figs S2–4).

In summary, we have developed an EOS for methane in nanoporous materials at supercritical temperature over a wide range of pressures. Our EOS successfully captures the key methane storage mechanisms, and links the methane storage behavior and microscopic properties including the varying critical properties, the gas intermolecular interaction, and the gas molecule-wall interaction. Our results demonstrate that our EOS is able to relate very well the methane storage behavior with each of the key physical parameters, including a pore size and shape and wall chemistry and roughness. Moreover, our EOS only requires one fitted parameter, and is simple, reliable and powerful in modeling the methane storage in nanoporous shale, screening the existing nanoporous materials and sketching images of the optimal candidates of nanoporous materials for methane storage in vehicular applications. Also, our EOS can be readily extended to other common gases (CO_2_, H_2_, N_2_, Ar and He), and generates new methods for the related fields of research, including gas separation^102^, carbon capture and storage^103^, membranes^104^ and catalysis^105^.

## Additional Information

**How to cite this article**: Wu, K. *et al*. Methane storage in nanoporous material at supercritical temperature over a wide range of pressures. *Sci. Rep.* **6**, 33461; doi: 10.1038/srep33461 (2016).

## Supplementary Material

Supplementary Information
