# Supplementary material for: Methane storage in nanoporous material at supercritical temperature over a wide range of pressures
Source: Sci Rep. 2016 Sep 15;6:33461. doi: 10.1038/srep33461 (PMC5024135; doi:10.1038/srep33461)
Supplement: Supplementary Information [file srep33461-s1.doc]

# **Methane storage in nanoporous material at supercritical temperature over a wide range of pressures**

Keliu Wu^1*^, Zhangxin Chen^1*^, Xiangfang Li^2^, & Xiaohu Dong^1,2^

^1^ The Department of Chemical and Petroleum Engineering, University of Calgary, Alberta T2N1N4, Canada;

^2^ Key Laboratory for Petroleum Engineering of the Ministry of Education, China University of Petroleum, Beijing 102249, China.

^*^**Corresponding authors:**

Name: Zhangxin Chen

Affiliation: The Department of Chemical and Petroleum Engineering, University of Calgary, Alberta T2N1N4, Canada.

Tel: (403) 220-7825

Email: [*zhachen@ucalgary.ca*](mailto:zhachen@ucalgary.ca)

Name: Keliu Wu

Affiliation: The Department of Chemical and Petroleum Engineering, University of Calgary, Alberta T2N1N4, Canada.

Tel: (403)966-3673

Email: [*wukeliu19850109@163.com*](mailto:wukeliu19850109@163.com)

## **Supplementary Figures**

| 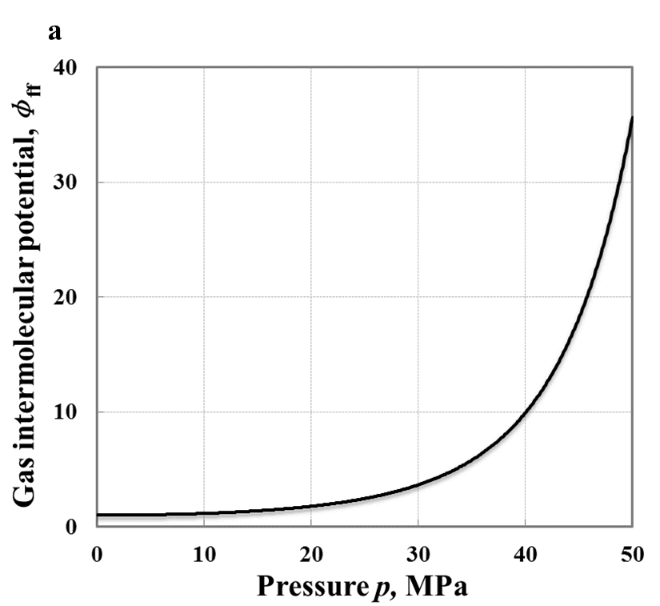 | 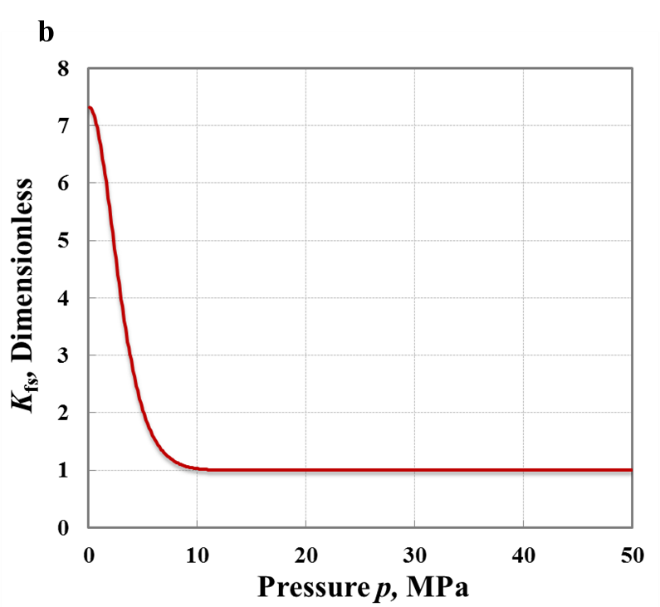 |
| --- | --- |
| **Supplementary Figure S1.** (**a**) Variation of the potential of methane molecules-molecules with pressure at *T*=298.15 K; (**b**) variation of the ratio of the interaction between methane molecules and nanopore walls to the methane intermolecular interaction with pressure for slit activated carbon pores with *H*=5 nm at *T*=298.15 K. | |

| 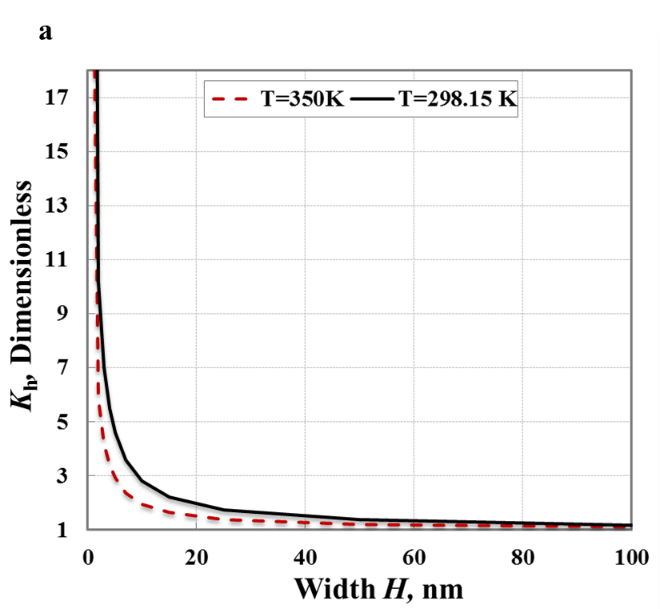 | 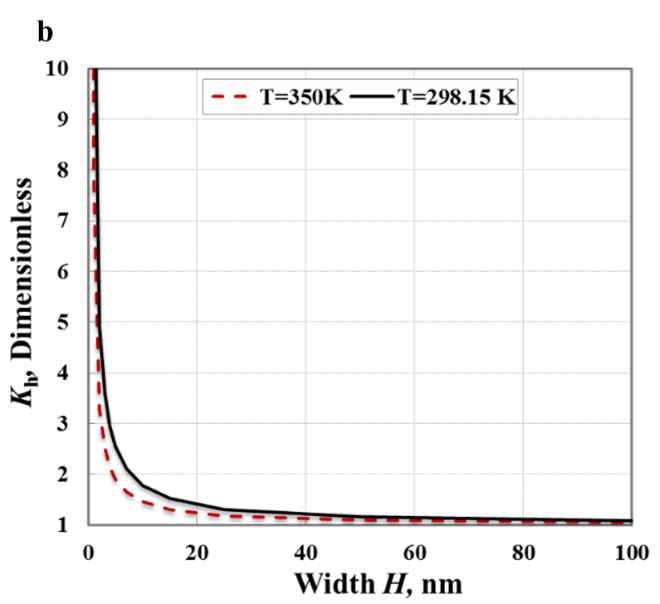 |
| --- | --- |
| **Supplementary Figure S2.** (**a**) The Henry law equilibrium constant for methane in slit pores with strong adsorption sites (activated carbon); (**b**) the Henry law equilibrium constant for methane in slit pores with weak adsorption sites (silica). | |

| 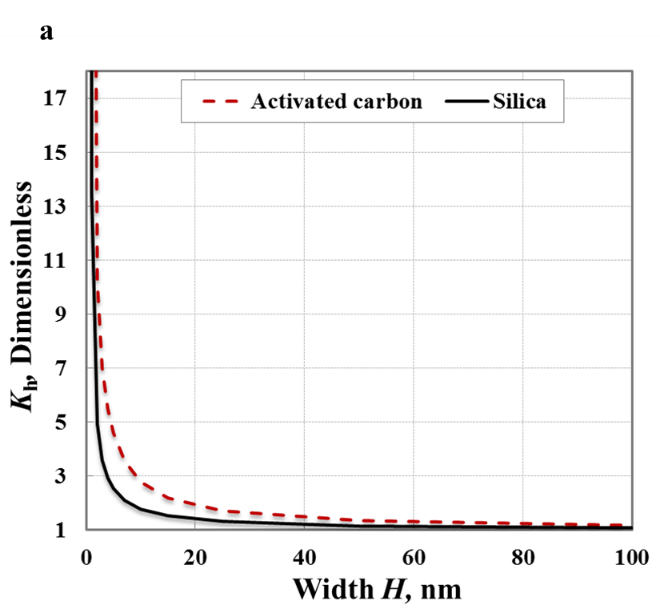 | 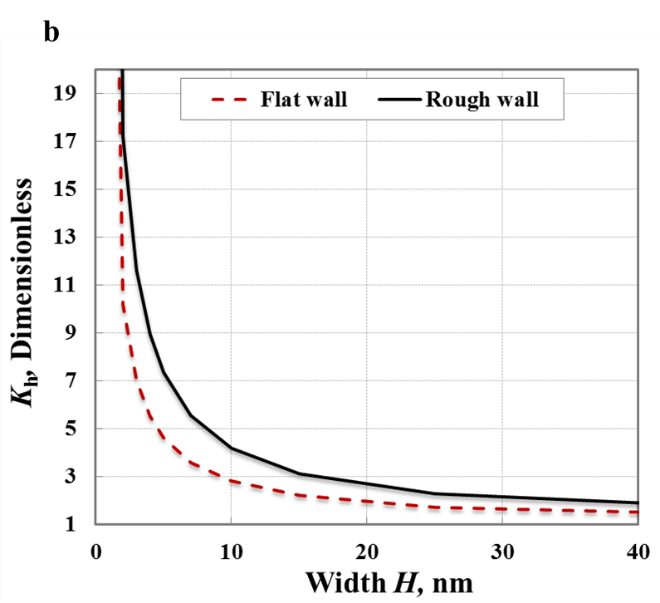 |
| --- | --- |
| **Supplementary Figure S3.** (**a**) Comparison of the Henry law equilibrium constants for methane in slit pores with strong adsorption sites (activated carbon) and weak adsorption sites (silica) at *T*=298.15 K; (**b**) comparison of the Henry law equilibrium constants for methane in slit activated carbon pores with flat and rough walls at *T*=298.15 K. | |

| **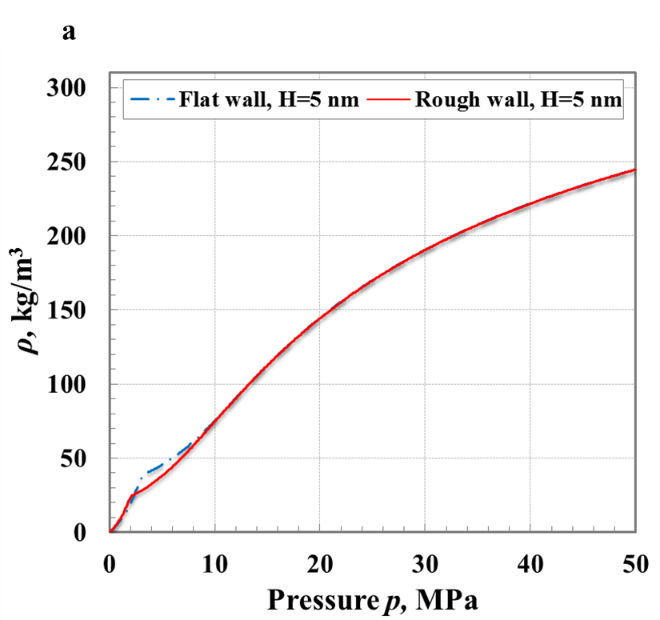** | **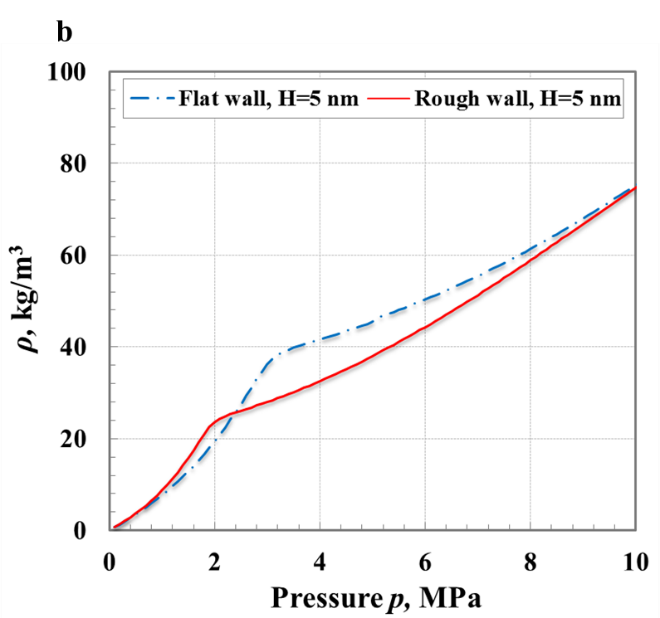** |
| --- | --- |
| **Supplementary Figure S4.** (**a**) Comparison of methane storage behavior in slit activated carbon pores with flat and rough walls for *H*=5 nm at *T*=298.15 K; (**b**) enlarged figure at low pressure. | |

## **Supplementary Tables**

**Supplementary Table S1.** The Lennard-Jones parameters for methane in our calculations.

| **Gas** | ***σ*_ff_ (nm)** | ***ε*_ff_ (K)** | **Ref** |
| --- | --- | --- | --- |
| Methane | 0.373 | 150 | 61 |

**Supplementary Table S2.** The Lennard-Jones parameters for pore walls with weak adsorption sites (silica).

| **Pore wall** | ***σ*_ss_ (nm)** | ***ε*_ss_/*k*_B_ (K)** | ***ρ*_s_ (nm^-2^)** | **Ref** |
| --- | --- | --- | --- | --- |
| Silica | 0.28 | 492.7 | 10.47 | S1 |

**Supplementary Table S3.** The Lennard-Jones parameters for pore walls with strong adsorption sites (activated carbon).

| **Pore wall** | ***σ*_ss_ (nm)** | ***ε*_ss_/*k*_B_ (K)** | | ***ρ*_s_ (nm^-2^)** | **Ref** |
| --- | --- | --- | --- | --- | --- |
| Activated carbon | 0.335 | 39.82 (flat wall) | 51.20 (rough wall) | 38.2 | S2, S3 |

**Supplementary Table S4.** Summary of the critical nanopore sizes in different fluid-nanopores systems. A critical nanopore size is defined as one above which fluid is expected to behave as a bulk fluid without being affected by the force field from nanopores walls.

| **Investigator** | **Method** | **Fluid-pore system** | **Critical pore size** |
| --- | --- | --- | --- |
| Vishnyakov et al. (2001)^86^ | Gibbs ensemble Monte Carlo method and the lattice gas model | Methane - slit graphite pores | 10-15 nm (strong field);  8-10 nm (weak field) |
| Travalloni et al. (2010)^S4^ | An extension of the  van der Waals equation of state | Fluid with the spherical molecules - cylindrical pores | 20 (the ratio of pore size to molecule size) |
| Singh and Singh (2011)^78^ | Grand-canonical transition-matrix Monte Carlo simulation | The square-well fluid - the hard and attractive cylindrical  pores | 50 (the ratio of pore size to molecule size) |
| Petropoulos and Papadokostaki (2012)^64^ | Advanced analytical surface transport theory | Gas - mesoporous slit pores | 20 (the ratio of pore size to molecule size) |
| Devegowda et al. (2012)^S5^ | Fitting formula on the basis of molecular simulation data | Shale gas - organic and inorganic pores | 10 nm |
| Ma and Jamili (2014)^S6^ | A modified Peng-Robinson equation of state on the basis of the simplified Local-Density theory | Methane - slit pores | 3 nm |
| Pitakbunkate et al. (2014)^82^ | Grand Canonical Monte Carlo simulation | Methane - slit graphite pores | 10 nm |

## **Supplementary Methods**

### **Critical properties**

A pore size and geometry and wall physical and chemical properties influence both the geometric constraint limiting the gas molecules number and the van der Waals forces exerted by nanopores walls. Thus, the critical properties of gas confined in nanopores are dependent on these factors. In addition, a layer thickness of the adsorbed gas reduces the space available for free gas, and further influences the critical properties. Using molecular dynamic simulations data (see Fig. 2 in the manuscript), the varying extents of the critical properties in nanopores can be determined and fitted by using some empirical formulas below.

**Cylindrical nanopores with strong adsorption sites**

A variation in the normalized critical temperature with the normalized nanopore size is expressed as

(4﹤*D/ δ* ≤50) (S1)

where *d*_a_ is the layer thickness of the adsorbed gas in nanopores and set to *δ* when single layer adsorption occurs.

**Cylindrical nanopores with weak adsorption sites**

A variation in the normalized critical temperature with the normalized nanopore size is expressed as

(3﹤*D/δ* ≤50) (S2)

A variation in the normalized critical pressure with the normalized nanopore size is expressed as

(3﹤*D/δ* ≤30) (S3)

**Slit nanopores with strong adsorption sites**

A variation in the normalized critical temperature with the normalized nanopore size is expressed as

(2﹤*H/δ* ≤40) (S4)

A variation in the normalized critical pressure with the normalized nanopore size is expressed as

(2﹤*H/δ* ≤30) (S5)

**Slit nanopores with weak adsorption sites**

A variation in the normalized critical temperature with the normalized nanopore size is expressed as

(2﹤*H/δ* ≤30) (S6)

A variation in the normalized critical pressure with the normalized nanopore size is expressed as

(2﹤*H/δ* ≤30) (S7)

Note that the critical pressure of methane in cylindrical nanopores with strong adsorption sites is not available in the literature. Thus, the critical pressure of methane in cylindrical nanopores with weak adsorption sites is adopted to calculate the density of methane in cylindrical activated carbon pores with *H*=5 nm at *T*=298.15 K (see Fig. 4c in the manuscript).

### **The potential of gas molecules and wall in a cylindrical pore**

For a cylindrical pore, the potential of gas molecules and the pore wall is expressed by using the well-known hypergeometric potential, as shown in equation (2) in the manuscript, where *ε*_fs_ and *δ*_fs_ are the Lennard Jones gas molecules-wall well depth and collision diameter, respectively, and are obtained by the Lorentz-Berthelot combination rules:

$\delta_{\text{fs}}=\frac{\delta_{\text{ss}}+\delta_{\text{ff}}}{2}$ (S8)

$\varepsilon_{\text{fs}}=\sqrt{\varepsilon_{\text{ss}}\varepsilon_{\text{ff}}}$ (S9)

*I*_1_ and *I*_2_ in equation (2) in the manuscript are expressed as^89^

$I_{1}=\frac{63\text{π}}{128D^{10}\left(1-\beta^{2}\right)^{10}}F\left(-4.5,-4.5;1;\beta^{2}\right)$ (S10)

$I_{2}=\frac{3\text{π}}{4D^{4}\left(1-\beta^{2}\right)^{4}}F\left(-1.5,-1.5;1;\beta^{2}\right)$ (S11)

where *F* is the hypergeometric function; *D* is the pore diameter; *β*=2*r*/*D*.

## **Supplementary Discussions**

### **The mechanism of a confinement effect**

In the area of nanopores studies, it is well established that the behavior of a fluid under confinement is significantly different from that of a bulk fluid because of non-negligible van der Waals forces, the geometric constraint, and a large specific area^S7^; these factors lead to fascinating surface-driven phase changes and various thermophysical and interfacial phenomena ^S7,85^. Gas in nanopores is divided into two groups, referred to as adsorbed gas and free gas, on the basis of whether a gas molecule passes through a force field from a wall or not^64^. For free gas, gas molecules are always under the influence of the force field due to the potential energy between the gas molecules and walls, and their moving trajectories are distorted by this force field^S8^, which leads to shortening the mean free path. Consequently, the free gas flow rate becomes smaller than the ideal gas flow rate, which is not affected by the force field^65^. For adsorbed gas, gas molecules have a relatively well-ordered and layered structure in the axial direction induced by the geometric constraint and the van der Waals forces exerted by nanopores walls. This structural difference causes a change in gas thermodynamic properties. The fascinating phenomenon is named a confinement effect, and it is somewhat equivalent to a pressure (and density) increase relative to the unconfined bulk gas due to the attraction exerted on gas molecules by the confining ‘‘walls’’^S9^.

### **The factors controlling a confinement effect**

A confinement effect obviously depends on several factors, such as physical and chemical properties of nanopores walls, nanopores geometry, nanopores size, gas properties, and temperature^76, 77^. Li et al. (2007) studied a water film with a thickness *h* < 2 nm, and found that its viscosity was higher than its bulk counterpart by four orders of magnitude on hydrophilic surfaces (mica and glass), whereas no size effect on hydrophobic surfaces (graphite) could be detected^S10^. In addition, the nanopores shape and size influence a confinement effect. The critical properties shifts for single component (C_1_, nC_4_ and nC_8_) are different in different pore shapes (slit or cylinder) ^85, 86^. The critical temperature decreases as the pore size decreases^84^. Hamada et al. (2007) also investigated the thermodynamics behavior of confined Lennard-Jones particles in slit and cylindrical pores systems using Grand Canonical Monte Carlo simulations, and indicated that fluid phase behavior, thermodynamic properties, and interfacial tension between the fluid and pore walls are affected by a pore size^77^. Puibasset (2005) also used the molecular simulation technique to investigate a confinement effect for cylindrical nanopores, and indicated that a chemical disorder of nanopores walls has a much stronger effect compared to a geometric disorder on the phase diagram of a confined fluid^S11^. Singh et al. (2011) concluded that a physical pattern of nanopores influences the vapour-liquid phase equilibrium and critical properties of a confined fluid^S12^. With the same nanopores, a different gas possesses a different confinement effect. Stronger solid-fluid interactions in a methane system lead to higher critical densities and lower critical temperature than in a neon system^86^. Temperature also influences a confinement effect; when temperature increases, the effect of the potential energy on the molecules in free gas decreases and the number of the adsorbed gas molecules on walls decreases^S8, 65^. In shale gas reservoirs, the adsorbed gas on nanopores walls also influences a confinement effect. As Singh et al. (2011) and Fisher and Nakanishi (1981) indicated, an adsorbed gas layer thickness further leads to a decrease in the critical temperature^S13^.

### **The extent of a confinement effect**

The extent of a confinement effect is determined by the ratio of fluid-fluid and fluid-wall interactions forces and a geometric constraint^86, S13^. The van der Waals forces have the longest range of all the colloidal forces, with a typical interaction length of 10 nm^64^. Hence, the confinement effect in a gas-nanopore system should have a wide range and large degree. A critical nanopore size is defined as one above which fluid is expected to behave as bulk fluid, without being affected by a force field from nanopores walls. There are varying critical nanopore sizes in different fluid-nanopores systems, as shown in **Table S4**. The confinement effect in nanopores is more noticeable for a narrower pore size and for a stronger adsorption field^86^. For microporous solids, the fluid molecule-wall interaction predominates^S14-S16, 76^. However, Trens et al.^S17-S19^ demonstrated that the fluid intermolecular interaction is enhanced, and plays a major role in mesoporous solids, but a confinement effect still occurs in mesoporous solids. Singh et al. (2010) concluded that when the ratio of the pore to molecule size is *H*/*δ* ≤ 2 or *H*/*δ* ≥20, the confinement effect is the same in the varying force field. However, for 2 ＜*H*/*δ*＜20, the confinement effect is sensitive to the varying force field^84^. Vishnyakov et al. (2001) also indicated that the diagrams for one- and two-layer pores are symmetrical and similar to the coexistence curve of the bulk phase; however, in three-layer pores, the coexistence curves are significantly different in the cases of strong and weak adsorptions^86^. The confinement effect is more prominent at higher fluid-wall interaction strength^84^. The ranges of the confinement effect are 10 - 15 nm (strong field) and 8 - 10 nm (weak field) in a methane-slit graphite pores system, respectively^86^. Due to the effect of the force field, both distributions of the adsorbed gas and free gas are very uneven across nanopores; especially at low temperature, the former is the densest near walls, and the latter increases in density away from walls; however, both become more uniform with an increasing temperature^S8^, which is because the ratio of the fluid-fluid and fluid-wall interactions increases with an increasing temperature, and the fluid-wall force plays a relatively weaker role in gas properties as compared to the fluid-fluid force.

### **The effects of confinement on fluid thermodynamic properties**

In confined nanopores, first the number of fluid molecules is limited; second, the nanopore wall potential is significant with increasing pore wall proximity to fluid molecules, and the fluid-wall and fluid-fluid interactions may become equally significant^S1^. The orientation of fluid molecules in confined nanopores is different from that of bulk fluid. Molecules in bulk fluid move randomly without specific orientation and direction. However, molecules in nanopores have a relatively well-ordered and layered structure in the axial direction of nanopores^82^. Furthermore, the distributions of the adsorbed gas and free gas are very uneven in the radial direction of nanopores^S7, S8^, as mentioned above. These structural and distribution differences can cause changes in fluid thermodynamic properties, and phase and transport behavior^S1, S8, 82^.

The varying fluid thermodynamic properties induced by a confinement effect include the critical pressure and critical temperature, density, viscosity, surface tension, compressibility factor, adsorption heat and effective van der Waals diameter of gas molecules^65^. Zarragoicoechea et al. (2004) showed that the shifts in the critical temperature are proportional to a nanopore size^80^. Normally, as the pore size decreases, the critical temperature and freezing/melting point tend to decrease^S20, S21^. Singh et al. (2011) also indicated that the critical temperature decreased continuously up to a certain minimum slit width, and then it became constant as the system approached a two-dimensional regime (the nanopore size is close to two to three molecular diameters). On the other hand, a shift in the critical pressure also monotonically decreases with a decrease in the slit width, and approaches a constant value with smaller pores. However, the critical density fluctuates with a decrease in the slit width^S12^. The obvious explanation for the decrease in the critical temperature is a weaker role of the fluid-fluid interaction in narrow pores as compared with the fluid-wall interaction. The confined fluid is denser than the bulk fluid, because the attractive potential of nanopores walls allows fluid molecules to adhere to the walls and also to interact strongly with each other. Consequently, the confined fluid condenses at lower pressure than the bulk fluid does. This phenomenon may shift the phase envelope to a lower critical temperature and pressure, and a different critical density^82, 86^. The critical pressure is more sensitive to a pore size than the critical temperature^79^. Akkutlu and Didar (2013) concluded that changes are about a 46% decrease in the critical pressure and an 18% decrease in the critical temperature for a gas mixture confined to pores of 3 nm width^83^. Pitakbunkate et al. (2014) concluded that a huge deviation of the confined methane density (about 70%) occurs in nanopores with a size of 1.0 nm due to a strong confinement effect of kerogen pores. However, it becomes less significant when the pore size increases as the deviation reduces to lower than 5% in nanopores with a size of 7.0 nm in the shale reservoir conditions^82^. Akkutlu and Didar (2013) indicated that gas density decreases with a decrease in the nanopores size, being an advantage for gas flow in shale gas reservoirs^83^. However, some investigators proposed that the density of hydrogen adsorbed and confined in carbon nanotubes could be 2.5–5.0 times greater than that expected for ‘‘close-packed’’ hydrogen^S13^. Hence, it is still a controversial issue. Jana et al. (2009)^87^ and Singh et al. (2010)^84^ showed that the critical density may decrease or increase depending on the wall–fluid interaction and extent of the geometric constraint. In our work, we have successfully explained and resolved this controversial issue. When the interaction between fluid molecules and walls dominates, the critical density increases. However, when the fluid intermolecular interaction dominates, the critical density decreases, and the interaction between fluid molecules and walls becomes a negative influence on the critical density. The results of the previous investigations about a confinement effect on fluid viscosity that are available in the literature are sometimes contradictory to each other^S22^. Among the previous experiments, Raviv et al. (2001) indicated that when water molecules were confined between two curved mica surfaces (the water film thickness is 0.4-3.5 nm), the effective viscosity remained similar to its bulk counterpart^S9^. Conversely, Li et al. (2007) studied water films with a thickness < 2 nm, and found that the effective viscosity was higher than its bulk counterpart by four orders of magnitude on hydrophilic surfaces (mica, glass), whereas no size effect on hydrophobic surfaces (graphite) could be detected^S10^. The surface tension is significantly influenced by a confinement effect^S23^. For nanopores, a stronger fluid-wall interaction introduces multilayers of fluid adsorbed on walls, which reduces the effective size of pores^S24, S25^ as well as the effects of the pore size on the surface tension, and surface tension has been known to decrease with a smaller pore size^S26^. The gas compressibility factor is a function of the critical pressure and temperature^S27^; hence, the gas compressibility factor is also influenced by a confinement effect. As the pore size decreases, the gas compressibility factor increases considerably at high pressure^83^. Derouane et al. (1987, 1988, 1989 and 1991) have quantified the role and importance of a confinement effect in microporous solids. As a result of the confinement effect, the heat of adsorption can be amplified by a factor of up to five, and the effective van der Waals diameter of the adsorbed molecules reduces substantially to about 85% of its value in the bulk gas phase^S14-S16, 76^. Therefore, molecules can enter and be adsorbed strongly in pores with a diameter substantially smaller than their van der Waals diameter in the gas phase^S13^.

It is worthy to note that a confinement effect causes changes in fluid thermodynamic properties, which can subsequently lead to large effects on fluid transport in nanopores^S28-S30^.

## **Supplementary References**

S1. Bhatia, S. K. & Nicholson, D. Modeling self-diffusion of simple fluids in nanopores. *J. Phys. Chem. B* **115,** 11700-11711 (2011).

S2. Levesque, D., Gicquel, A., Darkrim, F. L. & Kayiran, S. B. Monte Carlo simulations of hydrogen storage in carbon nanotubes. *J. Phys.: Condens. Matter* **14,** 9285 (2002).

S3. Frankland, S. J. V. & Brenner, D. W. Hydrogen Raman shifts in carbon nanotubes from molecular dynamics simulation. *Chem. Phys. Lett.* **334,** 18-23 (2001).

S4. Travalloni, L., Castier, M., Tavares, F. W. & Sandler, S. I. Critical behavior of pure confined fluids from an extension of the van der Waals equation of state. *J. Supercrit. Fluids* **55,** 455-461 (2010).

S5. Devegowda, D., Sapmanee, K., Civan, F. & Sigal, R. Phase behavior of gas condensates in shales due to pore proximity effects: implications for transport, reserves and well productivity. Paper presented at SPE Annual Technical Conference and Exhibition, San Antonnio, Texas, USA. Society of Petroleum Engineers. DOI: http://dx.doi.org/10.2118/160099-MS. 2012, October 8-10.

S6. Ma, Y. & Jamili, A. Using simplified Local Density/ Peng-Robinson equation of state to study the effects of confinement in shale formations on phase behavior. Paper presented at SPE Unconventional Resources Conference, The Woodlands, Texas, USA. Society of Petroleum Engineers. DOI: http://dx.doi.org/10.2118/168986-MS. 2014, April 1-3.

S7. Sparreboom, W., Van Den Berg, A. & Eijkel, J. C. T. Principles and applications of nanofluidic transport. *Nat. Nanotechnol.* **4,** 713-720 (2009).

S8. Nicholson, D. & Petropoulos, J. H. Influence of adsorption forces on the flow of dilute gases through porous media. *J. Colloid Interface Sci.* **45,** 459-466 (1973).

S9. Raviv, U., Laurat, P. & Klein, J. Fluidity of water confined to subnanometre films. *Nature* **413,** 51-54 (2001).

S10. Li, T.D., Gao, J., Szoszkiewicz, R., Landman, U. & Riedo, E. Structured and viscous water in subnanometer gaps. *Phys. Rev. B* **75,** 115415 (2007).

S11. Puibasset, J. Capillary condensation in a geometrically and a chemically heterogeneous pore: A molecular simulation study. *J. Phys. Chem. B* **109,** 4700-4706 (2005).

S12. Singh, S. K., Khan, S., Jana, S. & Singh, J. K. Vapour–liquid phase equilibria of simple fluids confined in patterned slit pores. *Mol Sim.* **37,** 350-360 (2011).

S13. Fisher, M. E. & Nakanishi, H. Scaling theory for the criticality of fluids between plates. *J. Chem. Phys.* **75,** 5857-5863 (1981).

S14. Derouane, E. G., André, J. M. & Lucas, A. A. A simple van der waals model for molecule-curved surface interactions in molecular-sized microporous solids. *Chem. Phys. Lett.* **137,** 336-340 (1987).

S15. Derouane, E. G., Andre, J. M. & Lucas, A. A. Surface curvature effects in physisorption and catalysis by microporous solids and molecular sieves. *J. Catal.* **110,** 58-73 (1988).

S16. Lambin, P., Lucas, A. A., Derycke, I., Vigneron, J. P. & Derouane, E. G. Van der Waals interaction at a material wedge. *J. Chem. Phys.* **90,** 3814-3822 (1989).

S17. Trens, P. *et al.* Study of n-hexane adsorption in MCM-41 mesoporous materials: a scaling effect approach of capillary condensation processes. *New J. Chem.* **28,** 874-879 (2004).

S18. Trens, P. *et al.* A macrothermodynamic approach to the limit of reversible capillary condensation. *Langmuir* **21,** 8560-8564 (2005).

S19. Trens, P., Tanchoux, N., Galarneau, A. & Fajula, F. New evidence of confinement effects in mesoporous materials and the definition of confined Pitzer acentric factors. *J. Phys. Chem. B* **109,** 16415-16420 (2005).

S20. Kanda, H., Miyahara, M. & Higashitani, K. Triple point of Lennard-Jones fluid in slit nanopore: Solidification of critical condensate. *J. Chem. Phys.* **120,** 6173-6179 (2004).

S21. Moore, E. B., de La Llave, E., Welke, K., Scherlis, D. A. & Molinero, V. Freezing, melting and structure of ice in a hydrophilic nanopore. *Phys. Chem. Chem. Phys.* **12,** 4124-4134 (2010).

S22. Chen, X. *et al.* Nanoscale fluid transport: size and rate effects. *Nano Lett.* **8,** 2988-2992 (2008).

S23. Tan, S. P. & Piri, M. Equation-of-state modeling of confined-fluid phase equilibria in nanopores. *J. Fluid Phase Equilib.* **393,** 48-63 (2015).

S24. Evans, R., Marconi, U. M. B. & Tarazona, P. Capillary condensation and adsorption in cylindrical and slit-like pores. *J. Chem. Soc., Faraday Trans. 2* **82,** 1763-1787 (1986).

S25. Derjaguin, B.V. & Churaev, N.V. Polymolecular adsorption and capillary condensation in narrow slit pores, *J. Colloid Interface Sci.* **54,** 157–175 (1976).

S26. Tolman, R. C. The effect of droplet size on surface tension. *J. Chem. Phys.* **17,** 333-337 (1949).

S27. Loebenstein, W. V. Calculations and comparisons of nonideal gas corrections for use in gas adsorption. *J. Colloid Interface Sci.* **36,** 397-400 (1971).

S28. Hirunsit, P. & Balbuena, P. B. Effects of confinement on water structure and dynamics: a molecular simulation study. *J. Phys. Chem. C* **111,** 1709-1715 (2007).

S29. Kalra, A., Garde, S. & Hummer, G. Osmotic water transport through carbon nanotube membranes. *Proc. Natl. Acad. Sci. USA* **100,** 10175-10180 (2003).

S30. Majumder, M., Chopra, N., Andrews, R. & Hinds, B. J. Nanoscale hydrodynamics: enhanced flow in carbon nanotubes. *Nature* **438,** 44-44 (2005).
